# Fatty Acid and Phytosterol Content of Commercial Saw Palmetto Supplements

**DOI:** 10.3390/nu5093617

**Published:** 2013-09-13

**Authors:** Kavitha Penugonda, Brian L. Lindshield

**Affiliations:** Department of Human Nutrition, Kansas State University, Manhattan, KS 66506, USA; E-Mail: kavithap@k-state.edu

**Keywords:** saw palmetto, serenoa repens, prostate, benign prostatic hyperplasia, prostate cancer, fatty acids, phytosterols, supplements

## Abstract

Saw palmetto supplements are one of the most commonly consumed supplements by men with prostate cancer and/or benign prostatic hyperplasia (BPH). Some studies have found significant improvements in BPH and lower urinary tract symptoms (LUTS) with saw palmetto supplementation, whereas others found no benefits. The variation in the efficacy in these trials may be a result of differences in the putative active components, fatty acids and phytosterols, of the saw palmetto supplements. To this end, we quantified the major fatty acids (laurate, myristate, palmitate, stearate, oleate, linoleate) and phytosterols (campesterol, stigmasterol, β-sitosterol) in 20 commercially available saw palmetto supplements using GC-FID and GC-MS, respectively. Samples were classified into liquids, powders, dried berries, and tinctures. Liquid saw palmetto supplements contained significantly higher (*p* < 0.05) concentrations of total fatty acids (908.5 mg/g), individual fatty acids, total phytosterols (2.04 mg/g), and individual phytosterols, than the other supplement categories. Powders contained significantly higher (*p* < 0.05) concentrations of total fatty acids than tinctures, which contain negligible amounts of fatty acids (46.3 mg/g) and phytosterols (0.10 mg/g). Our findings suggest that liquid saw palmetto supplements may be the best choice for individuals who want to take a saw palmetto supplement with the highest concentrations of both fatty acids and phytosterols.

## 1. Introduction

Saw palmetto (*Serenoa repens*) herbal supplements are commonly used by men to combat benign prostatic hyperplasia (BPH), a nonmalignant enlargement of the prostate. In addition to BPH, these supplements are commonly consumed by men diagnosed with prostate cancer. In 2007, 5.1% (~1.7 million people) of Americans 18 years of age or older reported using saw palmetto in the past 30 days [1]. In 2011, over $18 million in saw palmetto was sold in the United States, ranking third among herbal dietary supplements [2]. A systematic literature review of 11 studies identified saw palmetto supplements as one of five commonly used complementary or alternative medicine modalities by men with prostate cancer; use was 1.9%–24.9% [3]. Another study found that 13.8% of unaffected men whose brothers had been diagnosed with prostate cancer had taken saw palmetto at some point in their lives [4].

Saw palmetto’s putative mechanism of action is inhibition of 5α-reductase, the enzyme that converts testosterone into the more potent androgen dihydrotestoterone [5,6,7,8,9,10]. Studies that have found saw palmetto administration or treatment reduced androgen action support this belief [11,12,13,14,15]; however, not all studies have found that saw palmetto inhibits 5α-reductase or has anti-androgen action [16,17,18]. The believed bioactive components of saw palmetto are fatty acids and phytosterols. Saw palmetto extracts predominantly consist of fatty acids (~90%) and are unique compared with other extracts and vegetable and nut oils [19] in that they are a rich source of the saturated, medium-chain fatty acids laurate (12:0) and myristate (14:0) [20].

Several studies suggest that the fatty acids in saw palmetto extracts are responsible for its ability to inhibit 5α-reductase [21,22,23,24,25,26], but which fatty acid(s) is/are responsible for the inhibition varies. Some research suggests that saw palmetto phytosterols (β-sitosterol, campesterol, stigmasterol), inhibit 5α-reductase, prostate cancer cell/tumor growth, and/or BPH symptoms [27,28,29,30,31]; however, these phytosterols are not unique to saw palmetto extracts [32]. Nevertheless, a combination of fatty acids, phytosterols, and other bioactive components may be responsible for beneficial effects reported from saw palmetto supplements.

Saw palmetto extract decreased testosterone-induced prostate hyperplasia [15] in rats and prostate cancer progression in TRAMP mice [14]. A number of studies have found that saw palmetto supplements improve lower urinary tract symptoms (LUTS) in men suffering from BPH. The two largest, highest-quality BPH clinical trials [33,34], however, failed to find a benefit of supplementation, leading a systematic literature review to conclude that saw palmetto supplementation provides no benefit [35].

Although methodological issues or distinctions may be responsible for these conflicting findings, it is also possible that differences in the nutrient profiles of the saw palmetto supplements used in the studies influenced the results. In support of this possibility, different saw palmetto supplements exhibited varied effectiveness in inhibiting 5α-reductase activity and prostate cancer cell proliferation [36,37]. In addition, Wolsko *et al.* found that only 6/26 (26%) of published saw palmetto randomized-controlled trials reported performing quantitative analysis on the extract used [38]. This is important because the fatty acid content of saw palmetto supplements has been found to be −97% to +140% of stated dosages [39], and a separate study found that supplements mean free fatty acid percentages ranged from 40.7% to 80.7% [40].

Others have also measured fatty acid [15,20,41,42,43] or phytosterol content [42,44,45] of saw palmetto, but despite the reported differences in saw palmetto supplement contents, to the best of our knowledge, no one has characterized both the fatty acid and phytosterol contents of commercially available supplements or compared different supplement categories. Thus, we set out to characterize these saw palmetto supplement components, hypothesizing that we would find large differences in their quantities and composition.

## 2. Materials and Methods

### 2.1. Sample Procurement

Twenty commercially available saw palmetto supplements were procured from online and local sources. Standard reference material (SRM) 3251, a *Serenoa repens* extract, was purchased from the National Institute of Standards and Technology (NIST; Gaithersurg, MD, USA) to ensure the accuracy of the fatty acid and phytosterol analysis [20]. Supplements were classified into the following categories based on their physical properties: (1) liquids, (2) powders, (3) dried berries, and (4) tinctures (Table 1).

### 2.2. Sample Preparation

Representative samples from each category were prepared as follows. For liquids, 4–5 gel capsules were emptied into a microcentrifuge tube and gently vortexed. For powders, 4–5 powder capsules were emptied into a weigh boat and the contents were mixed thoroughly with a spatula to break up any lumps; tablets were ground to smooth powder using a mortar and pestle. Dried berries were ground using a coffee grinder until they reached a coarse powder texture. Tincture bottles were vortexed before taking a sample. SRM ampoules were vortexed and the contents were transferred to microcentifuge tubes. Samples, and SRM, were extracted and analyzed in duplicate. Care was taken to select the duplicates from the same lot number. All materials were from Fisher Scientific (Pittsburgh, PA, USA) unless noted.

### 2.3. Fatty Acid Extraction and Transesterification

Fatty acids were prepared for analysis using the one-step extraction-transesterification method [46]. Approximately 40 mg of SRM, 40 mg of liquid supplements, 150 mg of powder supplements, 150 mg of dried berry powder, or 250 mg of tinctures were placed into 15 mL tubes with Teflon-lined screw caps. These amounts were selected because they were estimated to contain ~10–50 mg of total fatty acids based on label information. Two milliliters of benzene containing 2 mg of the internal standard methyl tridecanoate (Sigma-Aldrich, St. Louis, MO, USA) and 3 mL of freshly prepared methanolic-HCl were added. The tubes were capped, vortexed, and incubated at 70 °C for 2 h in a water bath. After allowing them to cool to room temperature, 5 mL of 6% potassium carbonate and 2 mL of benzene were added, and the tubes were vortexed and centrifuged at 500× *g* for 5 min. The supernatant was carefully transferred to vials for analysis.

**Table 1:** Saw palmetto supplements’ names, manufacturing locations, and other label ingredients.

Saw Palmetto Supplement	Other Label Ingredients	Lot Number
**Liquids**		
Doctor’s Best (San Clemente, CA, USA)	Gelatin, glycerin, water	9I1215
GNC Herbal Plus (Pittsburgh, PA, USA)	Olive oil, gelatin, glycerin, caramel color, titanium dioxide	NI
Spring Valley Natural Foods (Springfield, MO, USA)	Olive oil, gelatin, glycerin, water	36994690904
Now Foods (Bloomingdale, IL, USA)	Extra virgin olive oil, gelatin, glycerin	11676001159
Solaray (Park City, UT, USA)	Extra virgin olive oil, gelatin, glycerin	132812
Saw Palmetto Harvesting Company (Lengby, MN, USA)	NI	NI
Jarrow Formulas (Los Angeles, CA, USA)	Pumpkin seed oil, vitamin E, gelatin, glycerin, water	43044J9
**Powders**		
Permixon (Pierre Fabre Medicament, Boulogne Cedex, France)	NI	G06167
Biochem (Country Life, Hauppauge, NY, USA)	Pygeum extract, magnesium stearate, calcium silicate, magnesium silicate, silica, cellulose	09G802C
Natures’ Way Products, Inc. (Springville, UT, USA)	Magnesium stearate, gelatin, glycerin	585612
Solaray (Park City, UT, USA)	Vegetable cellulose capsule	140401
GNC Saw Palmetto Formula (Pittsburgh, PA, USA)	Pumpkin seed meal powder, pygeum bark powder, lycopene, zinc, cellulose, dicalcium phosphate, povidone	5795KJ3613
**Dried Berries**		
Mountain Rose Herbs (Dried Berries, Eugene, OR, USA)	None	9498
Mountain Rose Herbs (Powdered Berries, Eugene, OR, USA)	None	9646
More than alive (Powdered Berries, Lobelville, TN, USA)	None	NI
**Tinctures**		
Oregon’s Wild Harvest (Sandy, OR, USA)	Organic alcohol, water	SB12159E01
GAIA Herbs, Inc. (Brevard, NC, USA)	Grain alcohol	6212054600
LA Naturals (Michigan City, IN, USA)	Grain alcohol 70%–80%, deionized water	7R44ALL
Teeter Creek Herbs (Ava, MO, USA)	Grain alcohol 40%–50%, distilled water	NI
Nature’s Answer (Hauppauge, NY, USA)	Vegetable glycerin, purified United States Pharmacopeia (USP) water	092469

NI: no information on the label.

### 2.4. Fatty Acid Methyl Esters (FAMEs) Gas Chromatography-Flame Ionization Detector (GC-FID) Analysis

Sample extracts were analyzed for fatty acid methyl esters using a Hewlett-Packard model 5890 GC (Hewlett-Packard, Palo Alto, CA, USA) with a SP-2560 capillary column (100 m × 0.25 mm × *d*_f_ 0.2 μm, Supelco, Inc., Bellefonte, PA, USA). Injection port and detector temperatures were maintained at 250 °C. Helium was used as the carrier gas at a flow rate of 1 mL/min. One microliter of sample was injected at a split ratio of 100:1. A temperature gradient was used with an initial oven temperature of 140 °C that increased to 200 °C at 2 °C/min, then to 245 °C at 4 °C/min, and the sample held at this temperature for 17 min. The total run time was 66 min. Fatty acid methyl esters (FAMEs) were identified by their retention times using the FAME standard mix (Supelco, Bellefonte, PA, USA) and quantified using Agilent ChemStation software [47]. Thirty-seven fatty acids were analyzed and quantified and contributed to the total fatty acid results reported, but only the six major fatty acids, laurate (C12:0), myristate (C14:0), palmitate (C16:0), stearate (C18:0), oleate (C18:1), and linoleate (C18:2), are reported individually. The mean percent coefficient of variance between duplicates for total fatty acids was 0.8%.

### 2.5. Phytosterol Extraction

Phytosterols were extracted using a method described previously with modifications [48]. Approximately 100 mg of SRM, liquid, powder, and dried berry supplements and 1000 mg of tincture supplements were weighed into separate 250 mL Erlenmeyer flasks. Forty milliliters of 0.3 M potassium hydroxide in methanol and 10 µL (1 mg/mL chloroform) of cholestanol (Steraloids, Inc., Newport, RI, USA) were added to each flask. The extracts were distilled at 80 °C using a condenser for 1 h with continuous stirring. After cooling the flasks to room temperature, 40 mL of double-distilled water was added. Twenty milliliters of hexane was then added, and the contents were transferred to a separatory funnel. The separatory funnel was carefully inverted, gently rotated, then allowed to stand for 5–10 min. The bottom white turbid aqueous layer was discarded retaining the clear upper hexane layer. The contents of the separatory funnel were then extracted by adding 20 mL of hexane twice. The clear hexane layers were collected and filtered through a glass funnel containing glass wool and sodium sulfate. Filtered hexane extracts were concentrated using a Brinkmann Buchi Rotavapor R110 (Buchi, Switzerland) at 50 °C, transferred to test tubes, then evaporated to dryness under nitrogen. The dried contents were redissolved in 1 mL of chloroform and stored at −80 °C for derivatization.

### 2.6. Phytosterol Derivatization

Phytosterols were derivitaized and analyzed by gas chromatography-mass spectrometry as described previously with modifications [49]. Two hundred microliters of extracted sample was used for derivatization. A smaller volume was used for samples that contained high levels of phytosterols. Samples were placed in 1.5 mL screw-cap GC vials (Agilent, Santa Clara, CA, USA), dried completely under nitrogen, and redissolved in 70 µL pyridine (99.5% extra dry with AcroSeal, Acros Organics, Geel, Belgium) for derivatization. The samples in pyridine were derivatized by adding 30 µL of *N*-trimethylsilyl-*N*-methyltrifluoroacetamide with 1% trimethylchlorosilane (MSTA + 1% TMCS Thermo Scientific, Bellefonte, PA, USA) and incubated at 50 °C for 60 min on a hot plate. Derivatized samples were completely dried down under nitrogen, redissolved in 100 µL of chloroform, and analyzed within 24 h of derivatization.

### 2.7. Phytosterol Gas Chromatography-Mass Spectrometry (GC-MS)

GC-MS was performed on an Agilent 6890N GC coupled to an Agilent 5975N quadruple mass selective detector. The GC was fitted with a DB-5MS capillary column (60 m × 0.25 mm × *d*_f_ 0.25 µm, Agilent Technologies, Santa Clara, CA, USA) with a 5% phenyl methyl siloxane stationary phase. Helium was used as the carrier gas at a column flow rate of 1 mL/min. The front inlet was operating at a pressure of 22.33 psi and 280 °C. An Agilent 7683 autosampler was used to inject 1 µL of the sample in the splitless mode. The GC temperature program was: initial temperature of 80 °C, increased 25 °C/min to 300 °C, then increased 3 °C/min to a final temperature of 325 °C, where it was held for 7 min. The total run time was 24.5 min. The mass spectrometer was operated in the electron impact (EI) mode at 70 eV ionization energy. The MS quad temperature was at 150 °C, and the MS source temperature was at 230 °C. The data were processed with Agilent Chemstation. The three major phytosterols campesterol, stigmasterol, β-sitosterol, were quantified and summed to calculate total phytosterol content. The mean percent coefficient of variance between replicates for total phytosterol was 10.8%.

### 2.8. Statistical Analysis

Data were analyzed using SAS 9.3 (SAS Institute Inc., Cary, NC, USA), with *p* < 0.05 considered significant. Natural logs were used to transform data that did not meet the assumptions of normality and/or homogeneity of variance. Differences in individual fatty acid and phytosterol quantities and percentages, total fatty acid and phytosterol quantities, and percentages among the four supplement categories were analyzed using one-way ANOVA with Tukey’s test. The variation between duplicates was assessed by dividing the standard deviation with duplicate mean and multiplying with 100 to calculate the mean percent coefficient of variation.

## 3. Results

### 3.1. Fatty Acid Quantities and Percentages between Supplement Categories

Fatty acid quantities and percentages of the SRM, liquid, powder, dried berry, and tincture saw palmetto supplements are shown in Table 2 and Table 3. Nature’s Answer was not included in the tincture means and was excluded from statistical analysis due to its negligible fatty acid content (0.1 mg/g). Oleate and laurate were the predominant fatty acids across the different supplement categories. Liquid supplements contained significantly higher quantities of total (908.5 mg/g) and individual fatty acids than powder, dried berry, and tincture supplements. Liquid supplements contained significantly higher percentages of oleate and total fatty acids and significantly lower percentages of laurate and myristate than the other supplement categories. The total fatty acid content of powder supplements (179.6 mg/g) was similar to dried berry (126.4 mg/g) but significantly higher than tincture supplements (46.3 mg/g). Powder supplements contained significantly higher quantities of palmitate and stearate than dried berries and tinctures and significantly higher quantities of linoleate and total fatty acids than dried berries. Powder supplements contained significantly higher palmitate and stearate percentages than the other supplement categories, and tinctures contained significantly higher percentages of laurate than liquid and powder supplements.

**Table 2:** Fatty acid quantities (mg/g) and composition (% of total fatty acids) in SRM, liquid, and powder saw palmetto supplements.

Supplement	Laurate (C12:0)		Myristate (C14:0)		Palmitate (C16:0)		Stearate (C18:0)		Oleate (C18:1)		Linoleate (C18:2)		Total Fatty Acid	
	mg/g	%	mg/g	%	mg/g	%	mg/g	%	mg/g	%	mg/g	%	mg/g	% *
**Liquid Extracts (*n* = 7)**														
Doctor’s Best	276.6	29.4	105.4	11.2	86.9	9.2	17.5	1.9	313.9	33.4	43.6	4.6	941.3	94.1
GNC Herbal Plus	82.9	8.9	33.8	3.6	94.0	10.1	25.4	2.7	582.3	62.4	59.1	6.3	933.8	93.4
Spring Valley Natural Foods	170.3	18.5	67.6	7.3	85.8	9.3	24.1	2.6	448.5	48.6	53.9	5.8	923.4	92.3
Now Foods	141.6	15.5	51.6	5.7	107.0	11.7	21.1	2.3	438.1	47.9	81.0	8.9	914.7	91.5
Solaray	141.9	15.6	57.1	6.3	90.9	10.0	21.2	2.3	476.0	52.1	55.6	6.1	913.0	91.3
Saw Palmetto Harvesting Company	254.9	28.3	98.5	11.0	84.4	9.4	16.2	1.8	312.5	34.7	34.1	3.8	900.0	90.0
Jarrow Formulas	127.2	15.3	47.5	5.7	93.1	11.2	32.5	3.9	230.2	27.6	248.2	29.8	833.4	83.3
**Mean**	**170.8 ^a^**	**18.8 ^a^**	**65.9 ^a^**	**7.2 ^a^**	**91.7 ^a^**	**10.1 ^a^**	**22.6 ^a^**	**2.5 ^a^**	**400.2 ^a^**	**43.8 ^a^**	**82.2 ^a^**	**9.3 ^a^**	**908.5 ^a^**	**90.9 ^a^**
**SEM**	**18.0**	**1.9**	**6.9**	**0.7**	**2.0**	**0.2**	**1.4**	**0.2**	**30.9**	**3.2**	**19.2**	**2.4**	**9.6**	**1.0**
**Powders (*n* = 5)**														
Permixon	96.7	29.5	38.5	11.8	30.5	9.3	5.6	1.7	111.7	34.1	11.5	3.5	327.3	32.7
Biochem	40.7	17.1	16.2	6.8	58.2	24.4	47.0	19.7	36.4	15.3	19.1	8.0	238.3	23.8
Natures’ Way	38.0	26.5	14.3	10.0	18.2	12.7	14.4	10.0	40.1	27.9	6.5	4.5	143.6	14.4
Solaray	40.5	33.7	14.8	12.3	11.9	9.9	2.5	2.1	34.2	28.5	6.1	5.1	120.1	12.0
GNC Saw Palmetto Formula	14.6	21.3	5.6	8.2	15.2	22.1	9.6	13.9	14.6	21.2	4.6	6.7	68.6	6.9
**Mean**	**46.1 ^b^**	**25.6 ^b^**	**17.9 ^b^**	**9.8 ^b^**	**26.8 ^b^**	**15.7 ^b^**	**15.8 ^b^**	**9.5 ^b^**	**47.4 ^b^**	**25.4 ^b^**	**9.6 ^b^**	**5.6 ^a^**	**179.6 ^b^**	**18.0 ^b^**
**SEM**	**9.0**	**2.0**	**3.7**	**0.7**	**5.7**	**2.1**	**5.4**	**2.3**	**11.1**	**2.2**	**1.8**	**0.5**	**30.7**	**3.1**
**Standard Reference Material**														
SRM 3251	259.9	26.3	10.6	10.6	86.6	8.5	16.9	1.7	331.1	34.6	50.2	6.0	983.6	98.4

* % of dry mass (weight/weight), different letters indicate significant differences (*p* < 0.05) between the four supplement categories in Table 2 and Table 3. Samples and SRM from the same lot, were extracted and analyzed in duplicate.

**Table 3:** Fatty acid quantities (mg/g) and composition (% of total fatty acids) in dried berry and tincture saw palmetto supplements.

Supplement	Laurate (C12:0)		Myristate (C14:0)		Palmitate (C16:0)		Stearate (C18:0)		Oleate (C18:1)		Linoleate (C18:2)		Total Fatty Acid	
	mg/g	%	mg/g	%	mg/g	%	mg/g	%	mg/g	%	mg/g	%	mg/g	% *
Dried berries (*n* =3)														
Mountain Rose Herbs (dried berries)	41.7	31.1	15.1	11.3	12.4	9.3	2.6	1.9	41.2	30.8	6.6	4.9	134.0	13.4
Mountain Rose Herbs (powdered berries)	37.1	28.6	15.6	12.0	12.6	9.7	2.1	1.7	43.9	33.9	4.8	3.7	129.7	13.0
More than Alive (powdered berries)	37.5	32.5	14.1	12.2	11.5	9.9	2.2	1.9	34.6	30.0	5.6	4.9	115.7	11.6
**Mean**	**38.8** **^b^**	**30.7** **^b,^^c^**	**14.9** **^b^**	**11.8** **^b^**	**12.2** **^c^**	**9.6** **^a^**	**2.3** **^c^**	**1.8** **^a^**	**39.9** **^b^**	**31.5** **^b^**	**5.7** **^b^**	**4.5** **^a^**	**126.4** **^b,c^**	**12.6** **^b,c^**
**SEM**	**0.9**	**0.7**	**0.3**	**0.2**	**0.2**	**0.1**	**0.1**	**0.1**	**1.7**	**0.8**	**0.3**	**0.3**	**3.5**	**0.3**
**Tinctures (*n* = 5)**														
Oregon’s Wild Harvest	28.4	29.2	10.6	10.9	8.7	9.0	1.7	1.8	32.0	32.9	4.5	4.6	97.3	9.7
GAIA Herbs	22.8	35.2	9.3	14.3	5.5	8.4	0.6	1.0	20.1	31.0	1.2	1.9	64.8	6.5
LA Naturals	5.1	40.2	1.7	13.4	1.1	8.6	0.2	1.4	3.0	23.1	0.7	5.6	12.8	1.3
Teeter Creek	3.7	36.5	1.2	11.6	0.7	7.4	0.1	1.3	2.4	23.6	0.3	2.7	10.1	1.0
Nature’s Answer **	0.0	0.0	0.0	0.0	0.0	0.0	0.0	19.3	0.0	0.0	0.0	0.0	0.1	0.0
**Mean**	**15.0** **^b^**	**35.3** **^c^**	**5.7** **^b^**	**12.5** **^b^**	**4.0** **^c^**	**8.3** **^a^**	**0.7** **^c^**	**1.4** **^a^**	**14.3** **^b^**	**27.6** **^b^**	**1.7** **^c^**	**3.7** **^a^**	**46.3** **^c^**	**4.6** **^c^**
**SEM**	**4.1**	**1.5**	**1.6**	**0.5**	**1.2**	**0.2**	**0.2**	**0.1**	**4.7**	**1.6**	**0.6**	**0.6**	**13.9**	**1.4**

* % of dry mass (weight/weight), ** Nature’s Answer was excluded from statistical analysis due to its negligible fatty acid content compared with other products. Different letters indicate significant differences (*p* < 0.05) between the four supplement categories in Table 2 and Table 3. Samples, and SRM from the same lot, were extracted and analyzed in duplicate.

### 3.2. Fatty Acid Quantities and Percentages within Supplement Categories

Within supplement categories, fatty acid quantities and percentages across the three dried berry supplements were fairly consistent (Table 2 and Table 3). Tinctures, on the other hand, varied widely, with total fatty acids ranging from 0.1 to 97.3 mg/g. There was less variability in total fatty acids in liquids (710.3–941.3 mg/g) and powder (68.6–327.3 mg/g) supplements; however, there were some notable trends. Among liquid supplements, Doctor’s Best and Saw Palmetto Harvesting Company contained higher laurate and myristate and lower oleate quantities. Jarrow Formulas contained much higher quantities of linoleate than the other liquid supplements. Among powder supplements, Biochem contained lower quantities of laurate and myristate levels but higher quantities of stearate and oleate.

### 3.3. Phytosterol Quantities and Percentages between Supplement Categories

Phytosterol quantities and percentages in SRM, liquid, powder, dried berry, and tincture saw palmetto supplements are shown in Table 4 and Table 5. Jarrow formulas (8.33 mg/g) and Biochem (22.80 mg/g) were not included in category means and were excluded from statistical analysis due to very high phytosterol content compared with the other supplements. β-Sitosterol was the predominant phytosterol (47.32%–79.48%) in all saw palmetto supplements. Liquid supplements contained significantly higher total (2.04 mg/g) and individual phytosterol quantities than powders, dried berry, and tincture supplements. Liquid supplements also contained a significantly higher percentage of β-sitosterol than the other supplement categories and a lower percentage of campesterol compared with powders and dried berry supplements. The total phytosterol content of powders (0.42 mg/g) and dried berries were similar, but powder supplements (0.33 mg/g) contained significantly higher phytosterol quantities than tincture supplements (0.10 mg/g). Dried berries also contained significantly higher stigmasterol quantities than tincture supplements. Dried berry supplements contained a significantly higher and lower percentage, respectively, of campesterol and β-sitosterol than the other supplement categories. Dried berry and tincture supplements also contained significantly higher percentages of stigmasterol than liquid and powder supplements.

### 3.4. Phytosterol Quantities and Percentages within Supplement Categories

Liquid and powder supplements had similar quantities of individual and total phytosterols, with the exception of Jarrow Formulas and Biochem, which contained much higher phytosterol quantities (Table 4 and Table 5). Dried berry supplements had similar quantities and percentages of individual and total phytosterols. All tincture supplements contained low phytosterols quantities.

### 3.5. Difference between Stated and Measured Total Fatty Acid Content

Stated total fatty acid content from supplement labels was only available for liquid supplements. These stated quantities are compared to measured content in Table 6. Measured total fatty acid content in five out of six liquid supplements was greater than or equal to the quantities stated on their labels. It is worth noting that one supplement whose measured content (833 mg/g) was below its stated amount, Jarrow Formulas, was only 7% lower than its stated content (850–950 mg/g).

**Table 4:** Phytosterol quantities (mg/g) and composition (% of total phytosterols) in SRM, liquid and powder saw palmetto supplements.

Supplement	Campesterol		Stigmasterol		β-sitosterol		Total Phytosterols	
	mg/g	%	mg/g	%	mg/g	%	mg/g	% *
**Liquids**								
Jarrow Formulas **	8.33	29.09	4.01	14.03	16.29	56.88	28.63	2.86
Doctor’s Best	0.54	19.21	0.24	8.53	2.04	72.26	2.83	0.28
Now Foods	0.29	12.56	0.13	5.66	1.92	81.78	2.35	0.23
Saw Palmetto Harvesting Company	0.34	21.05	0.16	10.09	1.12	68.86	1.62	0.16
Solaray	0.23	11.10	0.11	5.19	1.73	83.71	2.07	0.21
Spring Valley Natural Foods	0.15	7.20	0.11	5.52	1.80	87.28	2.06	0.21
GNC Herbal Plus	0.15	11.61	0.07	5.40	1.08	82.99	1.31	0.13
**Mean**	0.28 ^a^	13.79 ^a^	0.14 ^a^	6.73 ^a^	1.62 ^a^	79.48 ^a^	2.04 ^a^	0.20 ^a^
**SEM**	0.04	1.45	0.02	0.57	0.12	1.99	0.15	0.02
**Powders**								
Biochem **	22.80	30.30	19.75	26.55	32.55	43.15	75.11	7.51
Permixon	0.18	21.06	0.06	7.68	0.60	71.27	0.84	0.08
Nature’s Way	0.10	24.65	0.04	11.11	0.25	64.24	0.39	0.04
Solaray	0.07	23.89	0.03	9.30	0.20	66.80	0.30	0.03
GNC Saw Palmetto Formula	0.03	19.83	0.01	9.19	0.10	70.98	0.15	0.01
**Mean**	0.09 ^b^	22.36 ^b^	0.04 ^b,c^	9.32 ^a^	0.29 ^b^	68.32 ^b^	0.42 ^b^	0.04 ^b^
**SEM**	0.02	0.90	0.01	0.53	0.07	1.33	0.10	0.01
**Standard Reference Material**								
SRM 3251	0.41	19.69	0.18	8.57	1.48	71.74	2.06	0.21

* % dry mass (weight/weight), ** Jarrow Formulas and Biochem were excluded from statistical analysis due to their very high phytosterol content compared with other supplements. Different letters indicate significant differences (*p* < 0.05) between the four supplement categories in Table 4 and Table 5. Samples, and SRM from the same lot, were extracted and analyzed in duplicate.

**Table 5:** Phytosterol quantities (mg/g) and composition (% of total phytosterols) in dried berry and tincture saw palmetto supplements.

Supplement	Campesterol		Stigmasterol		β-sitosterol		Total Sterols	
	mg/g	%	mg/g	%	mg/g	%	mg/g	% *
Dried berries								
Mountain Rose Herbs (dried berries)	0.16	41.45	0.09	21.91	0.13	36.64	0.38	0.04
Mountain Rose Herbs (powdered berries)	0.09	27.93	0.06	17.41	0.17	54.66	0.32	0.03
More Than Alive (powdered berries)	0.08	27.28	0.07	22.06	0.15	50.66	0.30	0.03
**Mean**	0.11 ^b^	32.22 ^c^	0.07 ^b^	20.46 ^b^	0.15 ^b^	47.32 ^c^	0.33 ^b,c^	0.03 ^b^
**SEM**	0.02	3.30	0.01	2.86	0.01	5.16	0.03	0.00
Tinctures								
Oregon’s Wild Harvest	0.04	16.40	0.02	9.16	0.18	74.45	0.24	0.02
GAIA Herbs	0.03	17.95	0.02	11.65	0.11	70.40	0.16	0.02
LA Naturals Saw Palmetto drops	0.01	16.73	0.01	13.72	0.04	69.55	0.06	0.01
Nature’s Answer	0.00	21.12	0.00	25.23	0.01	53.65	0.02	0.00
Teeter Creek	0.01	22.27	0.00	14.71	0.02	63.02	0.02	0.00
**Mean**	0.02 ^b^	18.89 ^a,b^	0.01 ^c^	14.89 ^b^	0.07 ^b^	66.21 ^b^	0.10 ^c^	0.01 ^b^
**SEM**	0.00	0.83	0.00	1.86	0.02	2.47	0.03	0.00

* % of dry mass (weight/weight), different letters indicate significant differences (*p* < 0.05) between the four supplement categories in Table 4 and Table 5. Samples, and SRM from the same lot, were extracted and analyzed in duplicate.

**Table 6:** Stated, measured, and percentage difference in total fatty acid content in liquid saw palmetto supplements.

Product Name	Stated Content (mg/g)	Measured Content (mg/g)	% Difference
Doctor’s Best	900	941	+5
GNC Herbal Plus	850	934	+10
Spring Valley Natural Foods	850–950	923	+3
Now Foods	850–950	915	+2
Solaray	850	913	+7
Jarrow Formulas	850–950	833	−7

## 4. Discussion

We found great variability in total and individual fatty acid and phytosterol quantities and percentages in 20 commercial saw palmetto supplements. There was also a great deal of variability in the total and individual fatty acid and phytosterol quantities and percentages between the four different saw palmetto supplement categories. We believe we are the first to collect and analyze samples from these different supplement categories. Overall, we found that liquid supplements contained the highest fatty acid and phytosterol quantities, followed by powder, dried berry, and tincture supplements.

Fatty acid and phytosterol contents have been reported previously for SRM 3251 [20]. Because total fatty acid quantities were not reported, we multiplied the reported free fatty acid composition by the triglyceride to free fatty acid ratio. Our measured total fatty acid and total phytosterol contents (908.5 mg/g, 2.0 mg/g) are comparable with the calculated SRM 3251 values (983.6 mg/g, 2.4 mg/g) [20]. These results support that our extraction and analysis procedures were accurate in measuring these components. The total fatty acid percentages of liquid samples (83.3%–94.1%) are consistent with the values reported for hexane (98.7%–99.7%) [41], supercritical CO_2_ (96.1%–97.4%) [42], CO_2_ (92.2%) [43], ethanol (88.7%) [43] and lipid (92.5%) [15] saw palmetto extracts. The total fatty acid percentages are also consistent with the sum of the free fatty acids, esters, and glycerides (87.0%–95.3%) in 14 European saw palmetto extracts [40]. However, the total fatty acid percentages are higher than those reported for a saw palmetto ethanol extract (68.7%) [43].

Dried berry supplements contained 11.6%–13.4% fatty acids, which is similar to the levels reported in SRM 3250 (~15.8%), a saw palmetto berry, when calculated as described above [20]. Permixon, one of the powder supplements, has been analyzed previously and reported to contain 91.4% free fatty acids, esters, and glycerides [40]. This was far higher than the 32.7% fatty acid content that we measured. Our method may not extract fatty acids efficiently from powder samples and could be underestimating the fatty acid content. To the best of our knowledge, we are the first to measure tincture saw palmetto supplements. The levels of the major phytosterols (campesterol, stigamasterol, and β-sitosterol) in the saw palmetto supplements is consistent with quantities found in a variety of previously analyzed saw palmetto supplements [44] and is similar to the β-sitosterol content in a supercritical CO_2_ saw palmetto extract [42].

Ingredients added to saw palmetto supplements (Table 1) help explain the differences we found in fatty acid and phytosterol quantities and percentages. Most liquid, and a few powder, supplements contain added ingredients. For instance, among liquid supplements, only Doctor’s Best and Saw Palmetto Harvesting Company had no or a limited number of extra ingredients added to them. This probably explains why they have much higher laurate and myristate quantities. Jarrow Formulas, on the other hand, has a pumpkin seed oil base that is enriched in phytosterols, which explains its high linoleate and phytosterol quantities. The remaining four liquid supplements contained either olive oil or extra virgin olive oil (GNC Herbal Plus, Spring Valley Natural Foods, Now Foods, Solaray) which explains their higher oleate percentages compared to other liquid supplements. The powder supplements Nature’s Way and Biochem contain magnesium stearate, which explains their high quantities of stearate. Biochem also contains pygeum root extract, which is probably the reason why the supplement contains very high quantities of phytosterols [50].

Industrial preparative extraction methods may also help explain the differences in fatty acid and phytosterol quantities in the supplements. Saw palmetto supplements are usually extracted using solvents like hexane, ethyl alcohol, or supercritical CO_2_. All the tinctures were alcohol-extracted, but among liquid and powder supplements, only Doctor’s Best and Jarrow Formulas, which were extracted using supercritical CO_2_, stated how they were extracted. Thus, the unknown methods used to produce the supplements prevent us from understanding how extraction affects supplement fatty acid and phytosterol content. Other factors that might also contribute to the observed variation are batch differences and plant growing conditions.

We found that 83% of the liquid supplements contained greater than or equal to their stated fatty acid content. This is higher than the 33% of the measured saw palmetto supplements analyzed in a previous study [39]. Supplement companies that state their fatty acid content may be employing better manufacturing procedures and analyzing their products to ensure they contain the stated values.

## 5. Implications

Our findings suggest that liquid saw palmetto supplements are the best choice for individuals who want to take a saw palmetto supplement that has the highest concentration of both fatty acids and phytosterols. However, further research is needed to determine whether these supplements are indeed more efficacious.

## 6. Conclusions

We believe we are the first to characterize both fatty acid and phytosterol content of commercial saw palmetto supplements. We plan to determine the anti-androgen action of saw palmetto supplements with different nutrient profiles hoping that the understanding gained will explain the varied response to saw palmetto supplements seen in previous studies.
